# Three new species of the Chinese planthopper genus *Tetricodes* Fennah (Hemiptera, Fulgoroidea, Issidae, Parahiraciini)

**DOI:** 10.3897/zookeys.698.6101

**Published:** 2017-09-18

**Authors:** Zhi-Min Chang, Lin Yang, Jian-Kun Long, Xiang-Sheng Chen

**Affiliations:** 1 College of Animal Science, Guizhou University, Guiyang, Guizhou, 550025, P.R. China; 2 Institute of Entomology / Special Key Laboratory for Developing and Utilizing of Insect Resources, Guizhou University, Guiyang, Guizhou, 550025, P.R. China; 3 The Provincial Key Laboratory for Agricultural Pest Management of Mountainous Regions, Guizhou University, Guiyang, Guizhou, 550025, P.R. China

**Keywords:** Fulgoromorpha, issid, planthopper, morphology, taxonomy, distribution

## Abstract

The diagnostic characters of the Chinese planthopper genus *Tetricodes* Fennah, 1956 are redefined. Three new species of this genus, *T. ansatus* Chang & Chen, **sp. n.** (China: Guangxi), *T. parvispinus* Chang & Chen, **sp. n.** (China: Guizhou) and *T. similis* Chang & Chen, **sp. n.** (China: Guizhou) are described and illustrated. The female genitalia of the genus is described and illustrated for the first time. A checklist and key to the species of *Tetricodes* are given. The synonymy of *Tetricodes songae* Zhang & Chen, 2009 with *Tetricodes polyphemus* Fennah, 1956, **syn. n.**, is proposed.

## Introduction

The planthopper tribe Parahiraciini Cheng & Yang, 1991 (Hemiptera: Issidae) is a relatively small tribe distributed mainly in the Oriental region but also occurring in the Palaearctic region (Bourgoin 2016). In recent years, some genera and species were added or moved into this tribe (Gnezdilov and Wilson 2005; Meng et al. 2015; Wang et al. 2015, 2016; Gnezdilov and Hayashi 2016; Gnezdilov and Bourgoin 2016); to date, the tribe contains 19 genera and 68 species. Parahiraciini can be distinguished from other issid tribes by: body elongate ovate; forewings with claval suture; hindwings well developed, bilobed, and with a network of veins.

The genus *Tetricodes* was established by Fennah (1956) for *T. polyphemus* Fennah, 1956 as the type species from China (Hubei), with generic characters mainly based on a female specimen. Zhang and Chen (2009) transferred *Tetricodes* from the tribe Thioniini Melichar, 1906 to Parahiraciini and were first to recorded the male genitalia of the type species and one other species, *T. songae* from Guizhou, China. Subsequently, Chen et al. (2014) described another new species *T. anlongensis*. According to the frons with or without a light median line, Gnezdilov (2015) proposed that *T. polyphemus* in Zhang and Chen (2009) was a new species, *T. fennahi* Gnezdilov, 2015; up to date, the genus includes four species.

The main aim of this paper is to describe three new species of *Tetricodes* (Hemiptera: Issidae) from China (Guangxi, Guizhou), and propose a new species synonym. Also, type specimens are discussed, generic characteristics are redefined and a checklist and key to species of *Tetricodes* are provided.

## Materials and methods

The morphological terminology of the head and body follows Chan and Yang (1994), and the terminology of male and female genitalia follows Bourgoin (1993) and Gnezdilov (2002, 2003). Dry specimens were used for descriptions and illustrations. External morphology was observed under a stereoscopic microscope. All measurements are in millimeters (mm). The body measurements are from the apex of vertex to the tip of the forewing. The genital segments of the examined specimens were macerated in 10% KOH, washed in water and transferred to glycerine. Illustrations of the specimens were made with a Leica MZ 12.5 stereomicroscope. Photographs were taken with a KEYENCE VHX-1000C.

The type specimens of new and other species and other examined specimens are all deposited in the Institute of Entomology, Guizhou University, Guiyang, China (IEGU).

## Taxonomy

### Genus Tetricodes

Taxon classificationAnimaliaHemipteraIssidae

Fennah, 1956

Tetricodes Fennah, 1956: 513; Zhang and Chen 2009: 17; Chen et al. 2014: 116.

#### Type species.

*Tetricodes polyphemus* Fennah, 1956.

#### Diagnostic characters.

**Figures 1–6.**
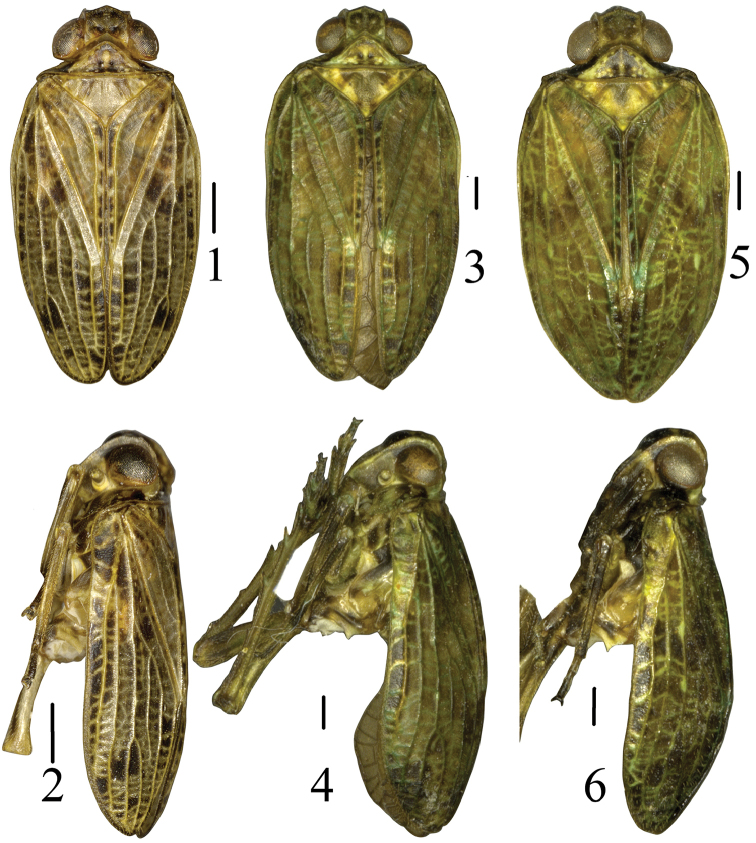
Dorsal habitus of *Tetricodes* species. **1–2** *Tetricodes ansatus* Chang & Chen, sp. n. **3–4** *Tetricodes parvulus* Chang & Chen, sp. n. **5–6** *Tetricodes similis* Chang & Chen, sp. n.. Scale bars = 0.5 mm.

**Figures 7–15.**
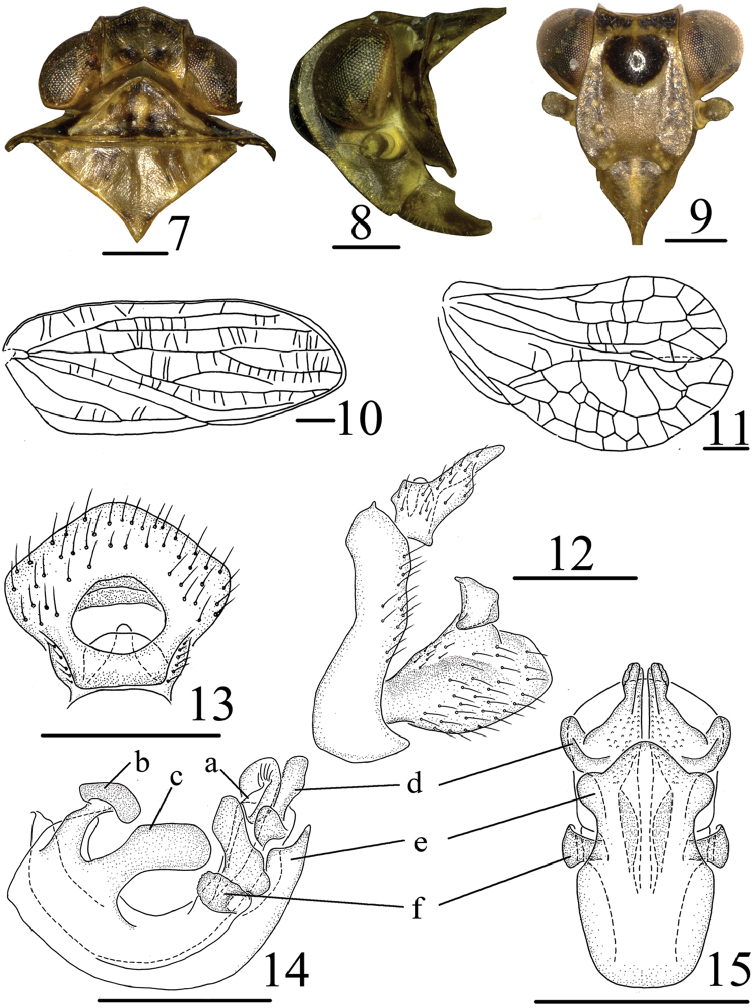
*Tetricodes ansatus* Chang & Chen, sp. n. **7** Head and thorax, dorsal view **8** Head and thorax, lateral view **9** Head, ventral view **10** Forewing **11** Hindwing **12** Male genitalia, lateral view **13** Anal segment, dorsal view **14** Aedeagus and phallobase, lateral view **15** Aedeagus and phallobase, ventral view. a–irregular lobes, b–bean-shaped process, c–ansate process, d–lateral lobe, e–ventral lobe, f–sheet-like processes. Scale bars = 0.5 mm.

Body slender beetle-like, oval. Width of head (Figs 1, 3, 5) including eyes narrower than pronotum. Vertex (Figs 7, 22, 31) with length in middle shorter than width at base, disc of vertex depressed, without median carina, with anterior margin obtuse-angled convex, posterior margin slightly concave. Frons (Figs 9, 24, 33) flat, median carina without or only present at base, big black hemispheroidal protuberance with light median line running from the upper margin, pale transverse band under the black protuberance. Clypeus triangular, broadly rounded, with median carina distinct or obscure. Rostrum surpassing mesotrochanters. Pronotum (Figs 1, 3, 5) short, with median carina obscure, lateral carinae not reaching to the posterior margin. Mesonotum (Figs 1, 3, 5) triangular, with median carina obscure, lateral carinae distinct. Hind tibiae each with 2–4 spines. Forewings (Figs 10, 26, 35) elongate, with length 2.3–2.6 times longer than maximum width, apical margin acutely rounded, ScP and Rp convergent near base, MP three or four branched, CuA simple, not forked; CuP present, Pcu and A_1_ uniting in clavus. Hindwings (Figs 11, 26, 35) well developed, as long as forewings, apical margin deeply incised into two big lobes, veins network-liked, anal lobe reduced.

#### Male genitalia.

Anal tube (Figs 13, 28, 37) short, irregularly oval or pentagonal in dorsal view. Anal style (paraproct and epiproct) (Figs 13, 28, 37) not surpassing anal tube. Pygofer (Figs 12, 27, 36) narrow, irregularly subquadrate. Genital styles (Figs 12, 27, 36) relatively short, without triangular prominence near dorsal margin before capitulum. Phallobase (Figs 14, 29, 38) with stout rod-like processes near base, lateral lobe splitting into two branches, with ventral lobe various. Aedeagus (Figs 14, 29, 38) with pair of foliaceous processes in lateral view.

#### Female genitalia.

**Figures 16–21.**
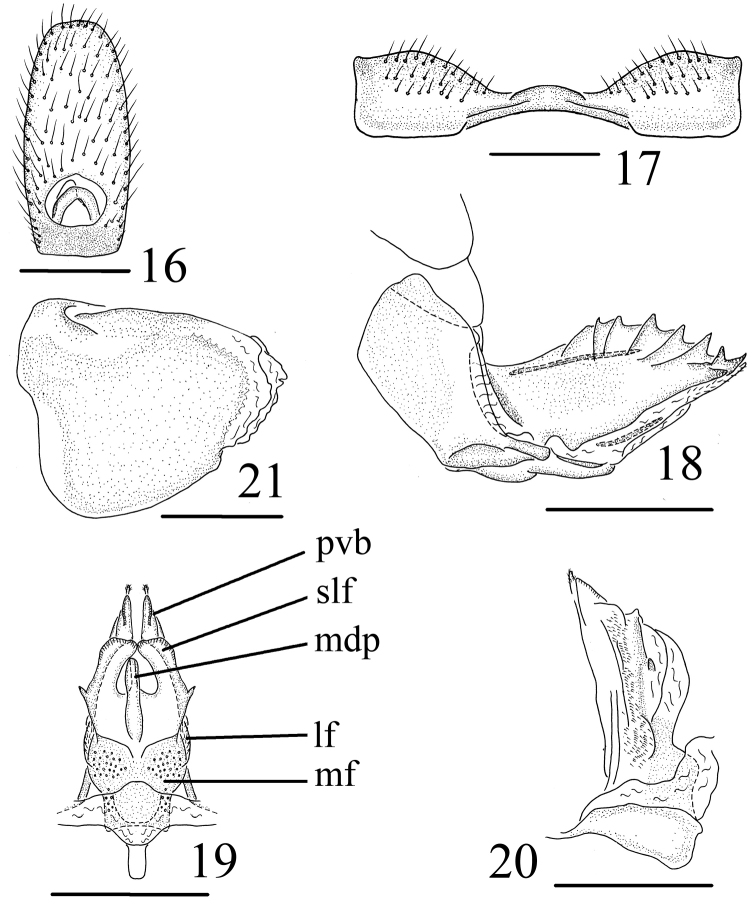
Female genitalia. *Tetricodes ansatus* Chang & Chen, sp. n. **16** anal segment, dorsal view **17** Sternum VII, ventral view **18** Anterior connective lamina of gonapophyses VIII, lateral view **19** Posterior connective lamina of gonapophyses IX, dorsal view **20** Posterior connective lamina of gonapophyses IX, lateral view **21** Gonoplacs, lateral view. Scale bars = 0.5 mm. Abbreviations. lf–lateral field of posterior connective lamina of gonapophyses IX; mdp–medial dorsal process; mf–medial field of posterior connective lamina of gonapophyses IX; pvd–posterior ventral lobes; slf–sublateral field of posterior connective lamina of gonapophyses IX.

Anal tube (Fig. 16) very long, truncated apically, anal style short, located at the base of anal tube. Hind margin of gonocoxa VIII with endogonocoxal lobe unobvious (Fig. 18), endogonocoxal process gradually narrowing (Fig. 18). Anterior connective lamina of gonapophyses VIII with 7 lateral teeth bearing 7 keels in lateral group and 3 apical teeth (Fig. 18). Gonoplacs (Fig. 21) without keels.

**Checklist of species of *Tetricodes* Fennah, 1956**

*Tetricodes anlongensis* Chen et al., 2014; China (Guizhou)

*Tetricodes ansatus* Chang & Chen, sp. n.; China (Guangxi)

*Tetricodes fennahi* Gnezdilov, 2015; China (Guizhou)

*Tetricodes parvispinus* Chang & Chen, sp. n.; China (Guizhou)

*Tetricodes polyphemus* Fennah, 1956; China (Guizhou, Hubei, Hunan, Yunnan)

*Tetricodes similis* Chang & Chen, sp. n.; China (Guizhou)

#### Key to species of *Tetricodes* (males)

**Table:** 

1	Anal tube concaved in apical margin	**2**
– Anal tube convexed in apical margin	**4**
2	Aedeagus with pair of small sheet processes at apical 1/3 in lateral view	**3**
– Aedeagus with pair of big sheet processes at apical 1/3 in lateral view (Fig. 38e)	***T. similis* sp. n.**
3	Anal tube with apical margin “V”-shaped concavely (see Chen et al., 2014: 2–63, Fig. H)	***T. anlongensis***
– Anal tube with apical margin “U”-shaped concavely (see Chen et al., 2014: 2–62, Fig. H)	***T. polyphemus***
4	Phallobase with two pairs of long processes (Fig. 14b, c)	***T. ansatus* sp. n.**
– Phallobase with pair of long processes	**5**
5	Anal tube irregularly pentagonal in dorsal view, the apical margin truncated (Fig. 28)	***T. parvispinus* sp. n.**
– Anal tube irregularly round in dorsal view, the apical margin arched (see Chen et al., 2014: 2–61, Fig. H)	***T. fennahi***

### Tetricodes anlongensis

Taxon classificationAnimaliaHemipteraIssidae

Chen, Zhang & Chang, 2014

Tetricodes anlongensis Chen et al., 2014: 120.

#### Material examined.

♂ (holotype), **China**: Guizhou, Anlong, Xianheping (24°58'N, 105°36'E), 28 Aug. 2012, S.-Y. Xu; 1♂1♀ (paratypes), same data as holotype.

#### Distribution.

China (Guizhou).

### Tetricodes ansatus

Taxon classificationAnimaliaHemipteraIssidae

Chang & Chen sp. n.

http://zoobank.org/88A2136F-A2FC-44DA-AAAB-ACB8EE2407A3

Figs 1–2 , 7–15 , 16–21

#### Type material.

Holotype: ♂, **China**: Guangxi, Nonggang (22°28'N, 106°57'E), 8 May 2012, H. Li; paratypes: 1♂, Guangxi, Nonggang, 8 May, 2012, Z.-H. Fan; 1 ♀, Guangxi, Nonggang, 6 May 2012, Z.-H. Fan.

#### Diagnosis.

This species is similar to *T. polyphemus*, but differs from the latter by the anal tube (Fig. 13) irregularly pentagonal in dorsal view, phallobase with two processes near basal dorsal margin (Fig. 14b, c) and aedeagus with pair of small sheet processes (Fig. 14f) at apical 1/3 in lateral view .

#### Description.

Body length: male 6.5–6.6 mm, female 6.7 mm; Forewing: male 5.3–5.5 mm, female 5.4 mm.

**Coloration.** General color brown, with irregular black mottling. Vertex with black speckles near base (Fig. 7). Eyes brown to black (Fig. 8). Frons (Fig. 9) with small black protuberance with light median line running from the upper margin, with narrow pale transverse clearer band under the black protuberance, to nearly the basal 1/3 of face, not reaching ventrally level of compound eyes, and pale spots near lateral margins. Clypeus brown with pale marks in middle line and rostrum brown. Forewings (Fig. 1) with irregular black mottling. Legs with tips of spines on hind tibiae and tarsi black.

**Head and thorax.** Head (Fig. 7) including eyes narrower than pronotum (0.76: 1.00). Vertex (Fig. 7) shorter in middle than the wide at base (0.57: 1.00), disc of vertex without median carina. Frons (Fig. 9) flat, with median carina only present at base, longer in middle than the widest breath (1.30: 1.00). Clypeus triangular, with distinct median carina and rostrum surpassing mesotrochanters. Pronotum (Fig. 7) with median carina obscure, lateral carina not reaching to the posterior margin. Mesonotum (Fig. 7) triangular, with median carina obscure, Hind tibiae each with 2 spines, spinal formula of hind leg 8–8–2 (male); 7–8–2 (female). Forewings (Fig. 10) elongate, 2.3 times as long as maximum breadth, ScP and Rp convergent near base, M four branched, MP_1+2_ bifurcates apically, MP_3+4_ bifurcates at middle part, CuA simple, not forked; CuP present, Pcu and A_1_ uniting in basal 2/3 of clavus. Hindwings (Fig. 11) with two big lobes, anal lobe reduced.

**Male genitalia.** Anal tube (Fig. 13) irregularly pentagonal in dorsal view, the widest breath in apical 1/3. Anal style (Fig. 13) short, located at the base 1/3 of anal tube. Pygofer (Fig. 12) narrow and curved in lateral view, irregular sub-quadrate. Genital styles (Fig. 12) short, without triangular prominence near dorsal margin before capitulum; capitulum of genital styles relatively short, subtrapezoidal on short neck. Phallobase with dorsal lobe cystiform at apical part, with irregular lobes in apical 1/4 in lateral view (Fig. 14a), bean-shaped process in basal dorsal margin (Fig. 14b), with big ansate process (Fig. 14c) in subbasal dorsal margin in lateral view; lateral lobe splitting into two stout branches, with stout sub-angular processes near apical (Figs 14d, 15d) ; ventral lobe short, not reaching to the tip of dorsal lobe, in ventral view ventral lobe with three lobes near apical part (Figs 14e, 15e). Aedeagus with pair of small sheet-like processes at apical 1/3 in lateral view (Figs 14f, 15f).

**Female genitalia.** Hind margin of sternum VII wide concavity, with curved process in middle in ventral view (Fig. 17). Anal tube (Fig. 16) sub-rectangular, truncated apically, longer in middle than the widest breadth (2.12: 1.00), anal style short, located at the basal 1/6 of anal tube. Anterior connective lamina of gonapophyses VIII with 7 lateral teeth bearing 7 keels in lateral group and 3 apical teeth (Fig. 18). Posterior connective lamina of gonapophyses IX (Figs 19, 20) long subtriangular, narrowing, median field with a slender shaft prominence (medial dorsal process) (Fig. 19); sublateral field with one triangular process on lateral margins; lateral field without process; ventroposterior lobes bent at obtuse angle (posterior ventral lobes) (Fig. 19). Gonoplacs (Fig. 21) without keels.

#### Etymology.

The name is derived from the Latin noun “ansatus”, meaning phallobase with big handle-shaped process in subbasal dorsal margin in lateral view.

#### Host plant.

Unknown.

#### Distribution.

China (Guangxi).

### Tetricodes fennahi

Taxon classificationAnimaliaHemipteraIssidae

Gnezdilov, 2015

Tetricodes fennahi Gnezdilov, 2015: 29.
Tetricodes polyphemus Fennah: Zhang and Chen 2009: 18; Chen et al. 2014: 118.

#### Material examined.

♂ (holotype), **China**: Guizhou, Leigongshan National Natural Reserve (26°28'N, 108°17'E), 2 Aug. 2004, F.-L. Xu.

#### Distribution.

China (Guizhou).

### Tetricodes parvispinus

Taxon classificationAnimaliaHemipteraIssidae

Chang & Chen sp. n.

http://zoobank.org/C7C7289C-ECE1-4204-A02E-A689DF19F191

Figs 3–4 , 22–30

#### Type material.

Holotype; ♂, **China**: Guizhou, Anlong, Xianheping (24°58'N, 105°36'E), 28 Aug. 2012, W.-B. Zheng; paratypes: 2♂♂, **China**, Guizhou, Anlong, Xianheping (24°58'N, 105°36'E), 28 Aug. 2012, W.-B. Zheng and J.-K. Long.

#### Diagnosis.

This species is similar to *Tetricodes anlongensis* Chen, Zhang & Chang, 2014, all from Guizhou: Anlong, Xianheping, but can be distinguished from the latter by the anal tube (Fig. 28) irregular pentagonal in dorsal view, the apical margin truncated, the widest breath in apical 1/3; phallobase with small process in dorsal margin of long lobe-shaped process at base (Fig. 29b).

#### Description.

Body length: male 6.4–6.8 mm; Forewing: male 5.3–5.5 mm.

**Figures 22–30.**
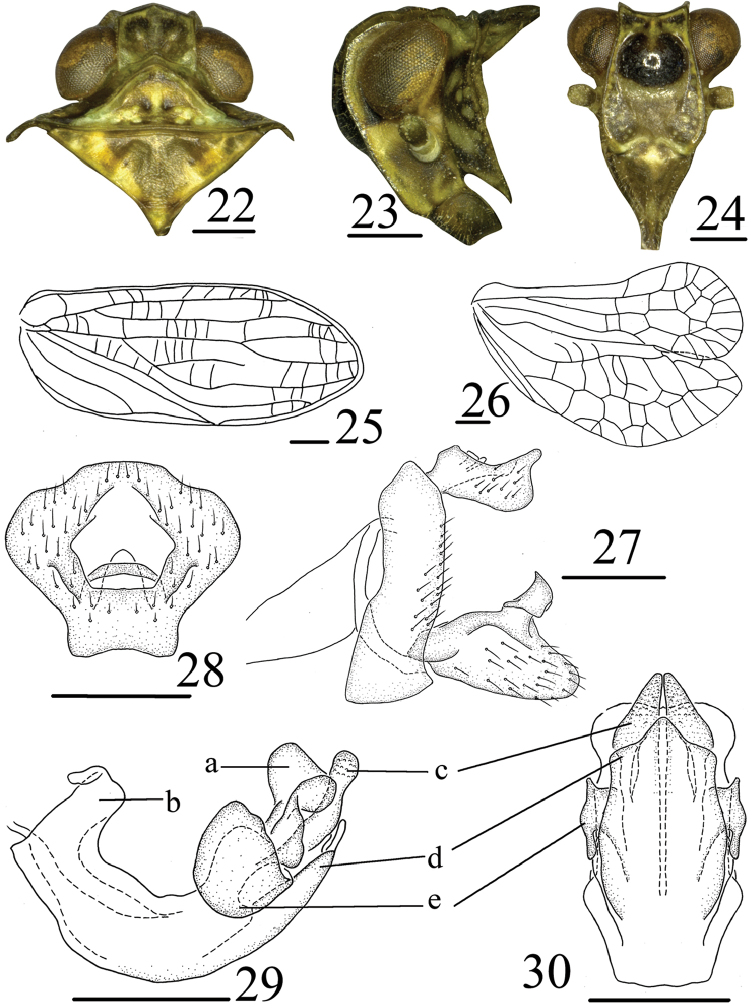
*Tetricodes parvulus* Chang & Chen, sp. n. **22** Head and thorax, dorsal view **23** Head and thorax, lateral view **24** Head, ventral view **25** Forewing **26** Hindwing **27** Male genitalia, lateral view **28** Anal segment, dorsal view **29** Aedeagus and phallobase, lateral view **30** Aedeagus and phallobase, ventral view. a–irregular lobes, b–long lobe-shaped process, c–lateral lobe, d–ventral lobe, e–sheet-like processes. Scale bars = 0.5 mm.

**Coloration**. General color yellowish green, with irregularly black mottling (Fig. 3). Eyes yellow to brown (Fig. 23). Frons (Fig. 24) with medium black protuberance with light median line running from the upper margin, with broad pale transverse clearer band under the black protuberance to nearly the basal 1/2 of face, reaching or surpassing ventrally level of compund eyes, and pale spots near lateral margins. Clypeus brown with yellow mark in base and rostrum brown. Pronotum (Fig. 22) with three pale vurrucae betwwen median carina and lateral carina. Forewings (Fig. 3) with irregularly black mottling near margins.

**Head and thorax**. Head (Fig. 22) including eyes narrower than pronotum (0.67: 1.00). Vertex (Fig. 22) shorter in middle than the wide at base (0.62: 1.00), disc of vertex without median carina. Frons (Fig. 24) flat, with median carina only present at base, longer in middle than the widest breath (1.34: 1.00). Clypeus triangular, with distinct median carina and rostrum surpassing mesotrochanters. Pronotum (Fig. 22) with median carina obscure, lateral carina not reaching to the posterior margin. Mesonotum (Fig. 22) triangular, with median carina obscure, Hind tibiae each with 4 spines, spinal formula of hind leg 8–10–2. Forewings (Fig. 25) elongate, 2.4 times as long as maximum breadth, ScP and Rp convergent near base, MP four branched, MP_1+2_ bifurcates apically, MP_3+4_ bifurcates at middle part, CuA simple, not forked; CuP present, Pcu and A_1_ uniting in basal 2/3 of clavus. Hindwings (Fig. 26) with two big lobes, anal lobe reduced.

**Male genitalia.** Anal tube (Fig. 28) irregular pentagonal in dorsal view, the apical margin truncated, the widest breath in apical 1/3. Anal style (Fig. 28) short, located in the middle of anal tube. Pygofer (Fig. 27) narrow and curved in lateral view, irregular subquadrate. Genital styles (Fig. 27) short, without triangular prominence near dorsal margin before capitulum; capitulum of genital styles relatively short, subtrapezoidal on short neck. Phallobase with dorsal lobe with irregular lobes (Fig. 29a) at apical part in lateral view, with small spine in dorsal margin of long lobe-shaped process in base (Fig. 29b); lateral lobe splitting into two stout branches, apical part subtriangular (Figs 29c, 30c); ventral lobe short, ventral view ventral lobe with three lobe near apical part, the apical lobe narrowed (Figs 29d, 30d). Aedeagus with pair of big sheet-like processes at apical 1/3 in lateral view (Figs 29e, 30e).

#### Etymology.

This species epithet is derived from combination of Latin root prefix “parv-” and “spine”, referring to the small spine in the dorsal margin of basal long process of aedeagus.

#### Host plant.

Unknown.

#### Distribution.

China (Guizhou).

### Tetricodes similis

Taxon classificationAnimaliaHemipteraIssidae

Chang & Chen sp. n.

http://zoobank.org/DC365C24-A0A6-4B5F-9D7B-7D11D7B730D1

Figs 5–6 , 31–39

#### Type material.

Holotype: ♂, **China**: Guizhou, Anlong, Xianheping (24°58'N, 105°36'E), 28 Aug. 2012, W.-B. Zheng.

#### Diagnosis.

This species can be distinguished from other *Tetricodes* species by the anal tube (Fig. 37) irregular circular, with sunken trilateral at apical margin in dorsal view; phallobase with dorsal lobe with irregular collar-shaped and long lobe-shaped process (Fig. 38a); lateral lobe with a stout lobe-like prominence and hook-shaped prominence in apical part in ventral view (Fig. 39c). Aedeagus with big sheet-like processes in lateral view (Fig. 38e).

#### Description.

Body length: male 5.8 mm, Forewing: male 4.9 mm.

**Figures 31–39.**
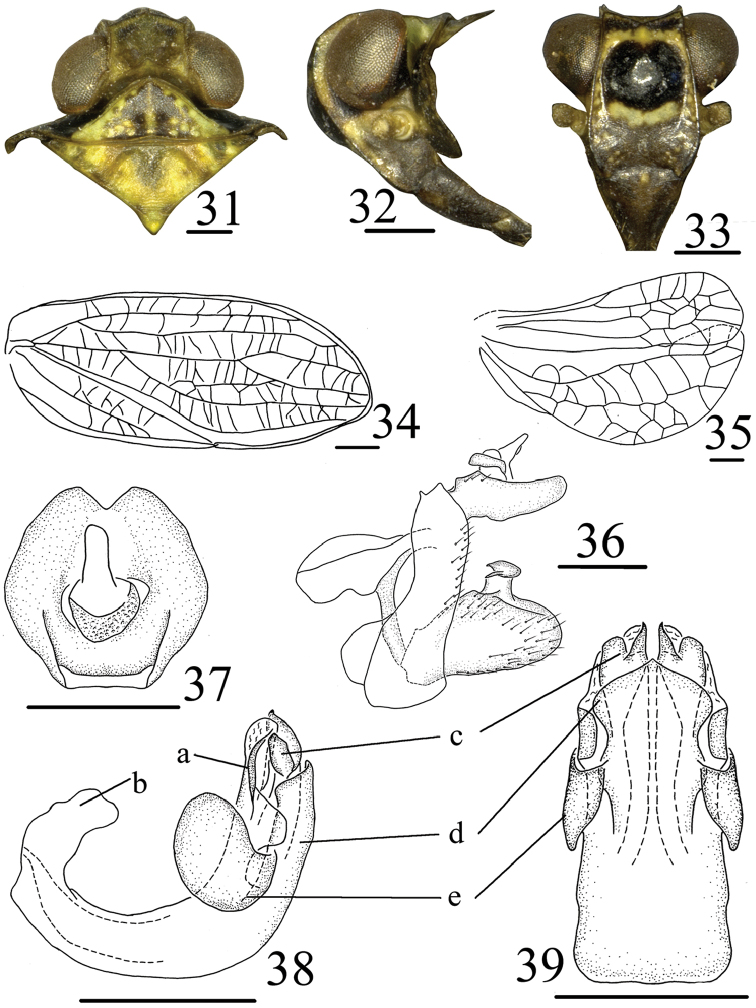
*Tetricodes similis* Chang & Chen, sp. n. **31** Head and thorax, dorsal view **32** Head and thorax, lateral view **33** Head, ventral view **34** Forewing **35** Hindwing **36** Male genitalia, lateral view **37** Anal segment, dorsal view **38** Aedeagus and phallobase, lateral view **39** Aedeagus and phallobase, ventral view. a–collar-shaped process, b–long lobe-shaped process, c–lateral lobe, d–ventral lobe, e–sheet-like processes. Scale bars = 0.5 mm.

**Coloration**. As in *T. parvispinus* Chang & Chen, sp. n., but clypeus black with yellow mark at base, rostrum brown and pronotum (Fig. 31) with four pale vurrucae between median carina and lateral carina.

**Head and thorax**. Head (Fig. 31) including eyes narrower than pronotum (0.67: 1.00). Vertex (Fig. 31) shorter in middle than the wide at base (0.63: 1.00). Frons (Fig. 33) longer in middle than the widest breath (1.30: 1.00). Clypeus triangular, with obscure median carina and rostrum surpassing mesotrochanters. Pronotum (Fig. 31) with median carina. Mesonotum (Fig. 31) with lateral carinae. Hind tibiae each with 4 spines, one of small spines near base, spinal formula of hind leg 8–10–2. Forewings (Fig. 34) elongate, 2.5 times as long as maximum breadth, ScP and Rp convergent near base, MP three branched, MP_1+2_ bifurcates apically, CuA simple, not forked; CuP present, Pcu and A_1_ uniting in basal 2/3 of clavus. Hindwings (Fig. 35) with two big lobes, anal lobe reduced.

**Male genitalia**. Anal tube (Fig. 37) irregular circular, with pitted triangular in apical margin in dorsal view, the widest breath in apical 1/2. Anal style (Fig. 37) long, located in the middle of anal tube. Pygofer (Fig. 36) narrow and curved in lateral view, irregular subquadrate. Genital styles (Fig. 36) short, with obscure triangular prominence near dorsal margin before capitulum; capitulum of genital styles relatively short, subtrapezoidal on short neck. Phallobase with dorsal lobe with irregularly collar-shaped process near apical part in lateral view (Fig. 38a), with long lobe-shaped process in dorsal margin in base (Fig. 38b); lateral lobe splitting into two branches, with stout lobe-like prominence and hook-shaped prominence in apical part in ventral view (Figs 38c, 39c); ventral lobe short, in ventral view ventral lobe with three lobe near apical part, the apical margin acute (Figs 38d, 39d). Aedeagus with pair of big sheet processes in apical 1/3 in lateral view (Figs 38e, 39e).

#### Etymology.

This new species is named for its similarity to the above new species.

#### Host plant.

Unknown.

#### Distribution.

China (Guizhou).

### Tetricodes polyphemus

Taxon classificationAnimaliaHemipteraIssidae

Fennah, 1956

Tetricodes polyphemus Fennah, 1956: 514.
Tetricodes songae Zhang & Chen, 2009 :19, figs 11-19, 23-27 ; Chen et al. 2014: 120, figs 2–62.

#### Material examined.

*Tetricodes polyphemus*: 1♀, **China**: Hubei Province, Lichuan, Maoba, Xingdoushan National Natural Reserve (30°01'N, 109°02'E), 4 Aug. 2010, X.- F. Yu; 2♂♂, 1♀, Hunan Province, Badagongshan National Natural Reserve (29°39'N, 109°49'E), 3 Aug. 2013, Z.-M. Chang and L. Qun. *Tetricodes songae*: ♂ (holotype), Guizhou Province, Leishan, Leigongshan National Natural Reserve (26°28'N, 108°17'E), 1 Aug. 2004, Q.-Z. Song; 2♂♂, 1♀ (paratypes), same data as holotype.

#### Diagnosis.

Frons with big black protuberance with light median line running from the upper margin, with broad pale transverse band under black protuberance, to nearly basal 2/3 of face, reaching or surpassing ventrally level of eyes, and pale spots near lateral margins of frons.

#### Distribution.

China (Guizhou, Hubei, Hunan, Yunnan).

#### Remarks.

The above synonymy is based on examination of the type specimens (male and female) of *Tetricodes songae* (from Guizhou Province) and the specimens of *T. polyphemus* collected from the type locality (Hubei Province, 1 female) and Hunan Province (male and female). Although Hunan, to which the locality of collecting the male material of *T. polyphemus* belongs, is different from the type locality (Hubei), the distance between the above localities is very close. The male genitalia of *T. songae* and *T. polyphemus* are identical.

## Discussion

The species of *Tetricodes* are very similar in colouration and external morphology. Based on black protuberance of frons without light median line, Gnezdilov (2015) considered that the species *T. polyphemus* treated by Zhang and Chen (2009) is another new species (*T. fennahi*). Actually, the light median line is variable in the examined specimens of all species. By contrast, in addition to male genitalic differences, the frons with black protuberance and pale transverse band, have certain value: small black protuberance and narrow pale transverse band (together in basal 1/3) in *T. ansatus* Chang & Chen; medium black protuberance and narrow pale transverse band in *T. fennahi* (together in basal 1/2); big black protuberance and broad pale transverse band (together in basal 2/3) in *T. polyphemus*; big black protuberance and broad pale transverse band in *T. anlongensis*, *T. parvispinus* Chang & Chen and *T. similis* Chang & Chen (together in basal 1/2–2/3).

## Supplementary Material

XML Treatment for Tetricodes

XML Treatment for Tetricodes anlongensis

XML Treatment for Tetricodes ansatus

XML Treatment for Tetricodes fennahi

XML Treatment for Tetricodes parvispinus

XML Treatment for Tetricodes similis

XML Treatment for Tetricodes polyphemus
